# Scaling life as an interspecies hallmark of aging

**DOI:** 10.1101/gad.353113.125

**Published:** 2025-08-01

**Authors:** Itamar Harel

**Affiliations:** Department of Genetics, Silberman Institute, Hebrew University of Jerusalem, Givat Ram, Jerusalem 9190401, Israel

**Keywords:** aging, interspecies, senescence

## Abstract

Across species, the “pace of life”—encompassing development, reproduction, and senescence—varies widely, yet the molecular mechanisms that regulate these interspecies trajectories of aging remain elusive. Even among vertebrates, a 1000-fold difference in life span is observed between species, ranging from several months in the turquoise killifish to half a millennium in the Greenland shark. As a relatively “young” area of investigation, aging research lacks the unifying conceptual frameworks that anchor more established disciplines, such as neuroscience. Therefore, current theories, which in some cases provide contradicting predictions, rely heavily on experimental data to mature. These contradictions not only define key outstanding questions but also illuminate fertile ground for transformative research.

## Abstract (precis)

In this Outlook, Harel connects evolution and aging by proposing that aging is a by-product of natural selection rather than a biological preset. Harel discusses recent research that supports the idea that temporal scaling and mortality risk underlie the evolutionary diversity of life span and the timing of reproductive maturity, with both being environmentally—and molecularly—tunable.

The “hallmarks of aging” (López-Otín et al. 2023) have provided a practical guide for mechanistic interventions. Each hallmark is defined by its ability to manifest during normal aging; in addition, experimental aggravation accelerates aging, and experimental amelioration retards aging. However, so far, the life span-extending effects of experimental interventions in model organisms do not account for the vast differences in longevity observed across species, which span several orders of magnitude. We propose temporal scaling of life as an addition to the existing framework—an experimentally tractable hallmark of aging that bridges evolutionary diversity with mechanistic insight.

## From physics to evolution

Why do some species live for decades or even centuries longer than others? Exploring this question has revealed that many physiological traits, such as organismal size, are closely linked to maximal life span. Allometric scaling laws have traditionally explained how body size influences physiology through physical principles such as geometry, metabolic scaling laws, and transport network efficiencies (Glazier 2025). Although these biophysical constraints set fundamental limits on size, energy use, and life span, they do not dictate how organisms allocate resources over time. Instead, evolutionary adaptations—reflected in life history strategies—govern the timing and investment in growth, reproduction, and survival. For example, despite similar allometric constraints, mice mature quickly and reproduce early, whereas elephants delay reproduction and invest in longevity. Such differences reflect evolutionary optimization, not physical necessity.

Organisms are under evolutionary pressure primarily to survive and reproduce. Once reproduction is achieved, the force of natural selection weakens (also known as the “selection shadow”). This idea is captured in two major theories (Williams 1957): the “mutation accumulation theory,” in which aging results from the buildup of deleterious mutations that only affect organisms postreproduction, and “antagonistic pleiotropy,” in which some genes provide reproductive or survival benefits early in life but have negative consequences later on. Thus, aging is not a predetermined biological program, but rather a by-product of natural selection. The evolutionary diversity of life span suggests that different species have adopted distinct strategies to manage these trade-offs (Jones et al. 2014).

A species’ external environment plays a crucial role in shaping its life span. If an organism is likely to die after a short time (due to predation or other unavoidable environmental hazards), it benefits from maturing quickly and reproducing early. This results in the “live fast, fade early” strategy seen in small, vulnerable species. Conversely, larger animals, which are less susceptible to predation, can afford to grow slowly and live longer. In some species, especially certain fish and reptiles, growth is indeterminate and fecundity is continued, which maintains selective pressure even in older individuals. Therefore, aging could be suppressed because reproduction and growth continue late into life. These strategies reveal how the distribution of mortality risk and reproductive timing influences not just interspecies life span variation (Li et al. 2023) but the onset and progression of aging-related decline.

## Integrating noncanonical models into experimental aging research

Comparative genomics has proven useful for exploring the genetic basis of complex traits, including extreme longevity—from the long-lived bowhead whales and rockfish to the “immortal” jellyfish to the naturally short-lived killifish. These studies have revealed that many longevity-linked genes fall into aging hallmarks and related pathways, including DNA repair and tumor suppression, insulin signaling, and inflammation (Li et al. 2023). In these studies, positive selection in long-lived species enhances these functions, whereas relaxed selection in short-lived species allows deleterious mutations to persist.

However, systematically investigating candidate genes identified through comparative genomics remains challenging. Although cellular models from long-lived species are useful for assessing specific hallmarks of aging, exploring intratissue communication or the scaling of life history traits typically requires whole-animal models. Classical models such as *Caenorhabditis elegans, Drosophila*, and mice continue to provide foundational insights into conserved molecular pathways of aging. However, these species represent an infinitesimally narrow slice of evolutionary strategies. Noncanonical models, particularly those exhibiting extreme life history plasticity, can reveal mechanisms that remain inaccessible in traditional systems.

For example, the freshwater polyp *Hydra* demonstrates remarkable longevity with no clear signs of senescence. In contrast, the African turquoise killifish (*Nothobranchius furzeri*) (Harel et al. 2015; Harel 2022; Astre et al. 2023) exhibits a unique adaptation at the opposite end of the spectrum: To survive through harsh environmental conditions (and elevated external mortality), puberty onset is accelerated, and its embryos can enter a state of suspended developmental program called diapause. During diapause, annual killifish embryos can remain in a dormant state for extended periods, sometimes exceeding their adult life span by an order of magnitude.

Many species can undergo different types of diapause, such as *C. elegans*, which can enter diapause as a postmitotic larvae. Biological age refers to the functional state of an organism, which, in these cases, does not appear to progress during diapause (and may even be reversed following exit from dormancy) despite the passage of chronological time. Therefore, this remarkable adaptation effectively decouples biological age from chronological time, allowing embryos/larvae to survive unfavorable environmental conditions without aging, challenging traditional linear models of aging.

These cases highlight how noncanonical models expose reversible, experimentally tractable states of aging and offer rare opportunities to decouple chronological age from biological age, thus offering fresh perspectives on the regulatory logic of aging across species.

## Disagreement between aging theories as a catalyst for discovery

### Dissecting evolutionary trade-offs using experimental sterility models

As current aging theories sometimes offer conflicting predictions (Gems 2022), they benefit from experimental data for refinement. These contradictions highlight key unresolved questions and may guide exciting research directions. For example, the “disposable soma theory” proposes that organisms allocate limited resources between reproduction and body maintenance, leading to aging as a consequence of prioritizing reproductive success over long-term somatic upkeep. Accordingly, this theory predicts that blocking reproduction may extend life span by reallocating somatic resources.

However, seminal experiments in *C. elegans* hermaphrodites have shown that although removal of either the germline or the entire gonad results in sterility, only germline-depleted hermaphrodites exhibit extended life span. This finding reveals a prolongevity signal originating from the remaining somatic gonad (Hsin and Kenyon 1999). However, it remained unknown how this apparent conflict is modulated in true males and females or whether it is conserved in vertebrates.

We addressed this by generating distinct forms of sterility in killifish (Moses et al. 2023, 2024). This was achieved by genetically blocking germline development at different stages, including postvitellogenic, previtellogenic, and complete germline ablation, thereby producing a stepwise reduction in reproductive investment. Our findings suggest that the presence of the germline itself, not merely its energetic cost, regulates somatic aging in a sex-specific manner (Moses et al. 2024). Identifying possible prolongevity signals emitted by the empty gonad and whether they are conserved in mammals could have exciting implications.

### Life history scaling and antagonistic pleiotropy

Similarly, few vertebrate studies have directly linked the molecular mechanisms of antagonistic pleiotropy to the regulation of life history traits within a normal, biologically relevant range (Flurkey et al. 2001; Stroustrup et al. 2016). For example, germline-depleted killifish (Moses et al. 2024) and Ames and Snell dwarf mice are long-lived but sterile, indicating that such mutations are unlikely to account for natural variation in life span and aging within wild populations.

In ongoing work (Moses et al. 2025), we directly manipulated a conserved puberty regulator identified through human and salmon GWAS. This intervention accelerated not only the timing of sexual maturation and growth but also the onset of aging phenotypes, effectively reproducing a faster “pace of life” within a single species. By shifting life history timing, this model reveals that developmental rate, reproductive timing, and somatic decline can be coordinately modulated by defined genetic inputs, suggesting that life history scaling may serve as a mechanistic entry point for dissecting the temporal regulation of aging.

The studies discussed above raise a set of fundamental yet unanswered questions at the intersection of life history theory and molecular aging biology. These emerging questions include the following:

1. Mechanistic universality: Which combination of aging hallmarks—established or emerging, and their interactions—shapes the strikingly diverse pace of life observed across species?
2. Temporal boundaries: When does aging begin within an organism's life history? How is its pace shaped by preceding developmental events? How flexible is the overall tempo of life?
3. Pace trajectories: Is there a universal pace of life, or do multiple trajectories coexist? Is it a linear process? To what extent do aging biomarkers shift in synchrony, or do they diverge?
4. Gene × environment: To what extent do ecological pressures modulate the pace of life? What insights can be gained from ecological niches that have fostered extreme adaptations, such as embryonic diapause, which appears to freeze biological time, or the “immortal” jellyfish, which may even reverse it?
5. Reprogramming logic: Following fertilization or during cellular reprogramming, the biological clock is reset. However, can we identify the specific set of factors that decouple dedifferentiation from rejuvenation during reprogramming?

Aging is often viewed through the lens of molecular hallmarks or stochastic decline, but comparative biology reveals a parallel truth: The pace of life is flexible, shaped by evolution, and experimentally tunable. By integrating noncanonical models with life history theory, we propose that temporal scaling of development, reproduction, and aging offers an underappreciated hallmark of aging biology—one that connects ecological context with cellular mechanisms. Recognizing and harnessing this axis may reveal new intervention points, expand our definitions of aging, and deepen our understanding of longevity across life.
